# Aripiprazole-Induced Neutropenia in a Seven Year-Old Male: A Case Report

**DOI:** 10.7759/cureus.1561

**Published:** 2017-08-11

**Authors:** Muhammad Hassan Majeed, Ali Ahsan Ali

**Affiliations:** 1 Department of Psychiatry, Quinnipiac University; 2 Brown University, Miriam Hospital

**Keywords:** antipsychotic, aripiprazole, blood dyscrasias, neutropenia

## Abstract

Blood dyscrasias are the widely known side effect of the second-generation antipsychotic medications. Aripiprazole rarely causes hematological side effects and it is considered relatively safe. We present the case report of a seven-year-old male who developed acute neutropenia a week after starting aripiprazole. His absolute neutrophil count (ANC) arose spontaneously once the medication was stopped. Clinicians should periodically check ANC in the patients taking aripiprazole as neutropenia could be lethal in extreme cases. To our knowledge, this is the first case report of leukopenia associated with aripiprazole in the child and adolescent population.

## Introduction

Blood dyscrasias are the widely known side effect of second-generation antipsychotic medications. In extreme cases, they could be lethal. Immune reactions being a direct toxic effect on bone marrow and increased destruction of cells are possible mechanisms in the pathophysiology of these blood dyscrasias [1]. An association of clozapine with neutropenia and/or agranulocytosis is well established, while aripiprazole is considered relatively safe with rare hematological side effects [2]. In 2009, the Food and Drug Administration (FDA) approved aripiprazole for the treatment of irritability associated with autism spectrum disorder in children aged six to 17 years [3]. In the child and adolescent population, aripiprazole is routinely used to treat various psychiatric and behavioral disorders [4]. Informed consent was obtained from the patient for this study.

## Case presentation

A seven-year-old African-American male was diagnosed with attention deficit hyperactive disorder and intellectual developmental disorder. He was stable on 5 mg Adderall XR (mixed Amphetamine Salts). After his parents separated, his behavioral problems increased at school. He was defiant, oppositional and had some aggressive outbursts towards his peers. The dose of Adderall XR was raised to 10 mg and then to 15 mg to treat irritability and aggression. His symptoms became worse with each dose increment. In an episode at school, he destroyed property and showed aggression towards the school staff. To ensure his own safety and that of others, he was admitted to the hospital.

During the course of the hospital stay, Adderall was stopped as it was thought to be worsening his irritability and aggression. To treat his aggression, 1 mg of aripiprazole at bedtime was started. The dose was increased to 2 mg on day three. There was no baseline clinical laboratory tests drawn during his inpatient hospital stay.

After four days of inpatient stay, the patient was discharged to an outpatient setting as he was no longer aggressive. He was seen in my office five days after he started aripiprazole. Complete blood count (CBC) and other investigations were ordered to monitor for metabolic syndrome. The results showed that the patients' white blood cell (WBC) count was normal with 3700 cells/µL and his absolute neutrophil count (ANC) was moderately low with 1600 cells/µL. After ruling out potential causes of neutropenia including common bacterial, virological and parasitical illness like hepatitis A, B, C, human immunodeficiency virus (HIV), malaria, salmonella or use of known drugs causing neutropenia, a phone call was made to the family to hold aripiprazole, as it could be causing the hematological abnormality. 

A phone call was made to primary care physician (PCP) to request the old medical records of the patient. A concern about low ANC count was communicated. After reviewing the records, PCP reported that the patients' previous blood report of six months ago showed ANC of 4000 cells/µL (within normal limits). A repeat complete blood count (CBC) was ordered three days after stopping aripiprazole and ANC rose to 2700 cells/µL and a week after to 4100 cells/µL (within normal limits). Follow-up at two weeks and four weeks showed an ANC of 4200 cells/µL and 4000 cells/µL respectively. Guanfacine extended-release (ER) 1 mg was started in place of aripiprazole to treat the patient's aggression.

## Discussion

Neutropenia induced by aripiprazole may be an idiosyncratic reaction. The FDA Adverse Event Reporting Event database found no significant association between aripiprazole and neutropenia in children [2]. Although, leukopenia is mentioned as a possible side effect of aripiprazole on its marketing brochure, very few cases have been reported in the clinical literature [5]. To our knowledge, there is no other case reported of neutropenia, associated with aripiprazole in the child and adolescent population. With low ANC in patients taking aripiprazole, physicians should consider the possibility of benign ethnic neutropenia in the patients with African or Middle Eastern descent [6]. 

## Conclusions

This case highlights the need for monitoring the ANC count to guard against the potential side effects of aripiprazole. There are clear guidelines to monitor for metabolic syndrome developed by the consensus of American diabetic, psychiatric, clinical endocrinologists, and study of obesity associations. One practical approach is to check CBC along with lipid profile and blood glucose when the patient initiates the treatment and then follow-up after starting aripiprazole.
